# Airborne Pesticides from Agricultural Practices: A Critical Review of Pathways, Influencing Factors, and Human Health Implications

**DOI:** 10.3390/toxics11100858

**Published:** 2023-10-13

**Authors:** Thirasant Boonupara, Patchimaporn Udomkun, Eakalak Khan, Puangrat Kajitvichyanukul

**Affiliations:** 1 Department of Environmental Engineering, Faculty of Engineering, Chiang Mai University, Chiang Mai 50200, Thailandudomkun.patchimaporn@gmail.com (P.U.); 2 Office of Research Administration, Chiang Mai University, Chiang Mai 50200, Thailand; 3 Civil and Environmental Engineering and Construction Department, University of Nevada, Las Vegas, NV 89154-4015, USA

**Keywords:** pesticide volatilization, spray drift, human health, air pollution, sustainable agriculture, integrated pest management

## Abstract

This critical review examines the release of pesticides from agricultural practices into the air, with a focus on volatilization, and the factors influencing their dispersion. The review delves into the effects of airborne pesticides on human health and their contribution to anthropogenic air pollution. It highlights the necessity of interdisciplinary research encompassing science, technology, public policy, and agricultural practices to effectively mitigate the risks associated with pesticide volatilization and spray dispersion. The text acknowledges the need for more research to understand the fate and transport of airborne pesticides, develop innovative application technologies, improve predictive modeling and risk assessment, and adopt sustainable pest management strategies. Robust policies and regulations, supported by education, training, research, and development, are crucial to ensuring the safe and sustainable use of pesticides for human health and the environment. By providing valuable insights, this review aids researchers and practitioners in devising effective and sustainable solutions for safeguarding human health and the environment from the hazards of airborne pesticides.

## 1. Introduction

The industrial revolution, while a remarkable success in terms of technological advancements, societal progress, and expanded services, has also resulted in significant air pollution. Anthropogenic air pollution poses a severe threat to global public health, causing approximately 9 million deaths annually [1]. Various large-scale human activities, such as the use of industrial machinery, power plants, combustion engines, and pesticides, contribute to the emission of air pollutants. Simultaneously, the world grapples with significant demographic shifts, climate change, and surging carbon dioxide emissions, all of which profoundly impact the realm of pesticides. With a burgeoning global population, the agricultural sector faces mounting pressure to increase food production, potentially intensifying pesticide use. Climate change-induced alterations in weather patterns and pest behaviors could reshape the types and quantities of pesticides required. Elevated carbon dioxide levels may disrupt crop–pest interactions, thereby influencing pesticide needs. These complex shifts in agricultural practices may also necessitate changes in labor demands and the adoption of innovative pesticide application approaches. It is worth noting that pesticides represent the largest category of synthetically produced substances. The term pesticide applies to insecticides, herbicides, fungicides, and other substances used to control pests, as well as plant regulators, defoliants, and desiccants. Approximately 2.6 million metric tons of active pesticide ingredients are employed globally [2]. Due to their persistence in the environment and bioaccumulative properties [3], global human, animal, and ecological health may be threatened by these sizable amounts. Consequently, pesticide application is strictly regulated. The transport of pesticides from crop-growing regions has resulted in widespread contamination, not only of soils, water bodies, and/or crops but also of the atmosphere via various pathways [4,5].

The atmosphere is probably the most critical medium for the long-distance transport of pesticides. Pesticides are found in the air in all three forms: solid, liquid, and gaseous. They potentially enter the atmosphere through different routes, including drift and evaporation during aerial spraying, volatilization from crops and agricultural soils, wind erosion of contaminated soils, and emissions from manufacturing and disposal processes [4,6,7,8]. Although pesticides and their alteration products mostly are semi-volatile organic compounds, they are not stable towards hydroxyl radicals and are prone to degradation upon exposure to these reactive species [9], which, in turn, enables airborne pesticides to be transported for long distances. For example, Lichota et al. [10] suggested that some persistent organic pollutants (POPs), such as -hexachlorocyclohexane (HCH) and hexachlorobenzene (HCB), which have been found in the endangered marmot (*Marmota vancouverensis*) from subalpine meadows on Vancouver Island, Canada, might be associated with the long-range atmospheric transport across the Pacific Ocean from the notion of distant (e.g., Asian) sources of these pesticides [10].

Consequently, understanding the sources, transport, and impacts of airborne pesticides is critical to assessing their ecological and health risks. It is also essential to investigate the path of pesticide dispersion into the air and to limit the adverse effects of pesticides on the environment and human health. To our knowledge, a systematic investigation of the impacts of environmental pesticide pollution from agricultural activities via the volatilization process on air quality and human health has not been conducted. Therefore, this critical review article aims to understand how pesticides from agricultural practices enter the air, particularly through volatilization, and examines their impact on human health and the environment. It critically assesses the environmental and human health impacts of commonly used pesticide spraying methods and proposes effective strategies to minimize pesticide exposure. Additionally, the study includes a preliminary cost analysis of mitigation strategies tailored to reduce associated environmental and health risks. The review concludes with research recommendations focused on mitigating pesticide dispersion and safeguarding ecosystems and human populations from the adverse effects of airborne pesticides.

## 2. Methods

This study conducted a systematic literature review following the Preferred Reporting Items for Systematic Reviews and Meta-Analyses (PRISMA) guidelines [11]. The review employed various search terms to retrieve articles from the ScienceDirect and PubMed databases, focusing on topics such as pesticide application, dispersion, airborne pesticide emissions and volatilization, pesticide properties, human health risks, environmental risks, and mitigation strategies. Only English-language articles were included, with a primary focus on more recent findings from 2005 to 2023, but also referencing notable pre-2005 work and documents from environmental and health organizations (e.g., EPA, NIOH, WHO, etc.). Screening of relevant papers was based on titles, abstracts, and keywords, excluding non-original works, seminar or conference abstracts, viewpoints, opinions, and encyclopedias. A total of 214 different articles were selected, and their full texts were thoroughly examined and synthesized in this review.

## 3. Existence and Dispersion of Pesticides in the Atmosphere

### 3.1. Existence of Pesticides in Outdoor Air

The potential adverse health effects on humans from pesticide exposure have caused significant public concern for a long time. Many studies assessed and reported their contamination in water and soil, while less work documented their existence in air. This is due to the nature of pesticides as low-level contaminants in the air, normally in the range of pg/m^3^ to ng/m^3^. Pesticides can be detected in the air in the form of solids, gases, and liquids [12]. During spraying in agricultural areas, about 30–50% of most pesticides are dispersed into the air [13] through drift (by the wind) and evaporation. Subsequently, pesticides are vaporized from soil and plants, degraded, and photolyzed prior to entering the atmosphere. Two factors prominently stand out among the contributors to pesticide impact: the physical and chemical properties of pesticides, and the surrounding environmental conditions [14].

Many pesticide groups (organochlorine insecticide, organophosphate insecticide, herbicide, and fungicide) were detected in the outdoor air in many countries, such as France, Spain, Austria, Italy, Germany, Republic of Korea, China, Pakistan, South Africa, and the U.S. The concentration ranges of pesticides in outdoor air are listed in Table 1. Over the past 15 years, the predominant organochlorine insecticides have included 4,4′-dichlorodiphenyldichloroethane (DDD), 4,4′-dichlorodiphenyldichloroethylene (DDE), and α-endosulfan. Meanwhile, within the organophosphate category, chlorpyrifos and diazinon have been frequently identified. Additionally, a range of herbicides and fungicides has been detected in fine airborne particulate matter (PM 2.5) [6,15,16], with glyphosate being the most commonly detected fungicide, and chlorothalonil and folpet prevailing among the fungicides.

### 3.2. Pesticide Properties and Dispersion Behavior

Several pesticide properties affect their ability to volatilize and enter the atmosphere as a gas [35]. The physicochemical properties of the compound, specifically vapor pressure, water solubility (and thus Henry’s law constant), and adsorption coefficient, substantially affect the atmospheric distribution of pesticides.

The most important physical property for pesticide transmission through the air is vapor pressure. Because pesticides can readily transform from a liquid or solid to a gaseous state, pesticides with higher vapor pressures are more likely to volatilize [36]. Bidleman [37] pointed out that compounds with a vapor pressure higher than 10^−2^ Pa are principally found in the gas phase, while those with a vapor pressure lower than 10^−5^ Pa are almost exclusively presented in the atmospheric particulate phase. Due to these, pesticides, which have a vapor pressure between the two numbers, could be found in both phases. A high correlation between the volatilization flux during the first hours after application and the vapor pressure was detected in many studies [38,39,40]. 

Guth et al. [41] analyzed the data from 80 different pesticides under controlled conditions and showed that vapor pressure was the best predictor of volatile losses from soil and crops. Likewise, a very high regression correlation (R^2^ = 0.98) between the evaporation rate and vapor pressure of 82 different pesticide active ingredients and co-formulants, ranging in vapor pressure from 0.0001 to > 30,000 Pa, was reported by van Wesenbeeck et al. [39]. Lichiheb et al. [42] developed a compartmental approach with the physicochemical properties of pesticides (chlorothalonil, fenpropidin, and parathion) to identify the dissipation processes of pesticides. The high sensitivity of the model was due to vapor pressure, and in addition to that, the octanol–water partition (K_ow_) coefficient represents an important physicochemical property influencing pesticide volatilization from plants. However, in practical situations, the “effective” vapor pressure might be lower than the “pure” vapor pressure due to pesticide interactions with the soil surface. Nevertheless, vapor pressure remains a valuable parameter for screening compounds in a tiered risk assessment scheme [43]. Henry’s law constant (HLC) is an additional essential parameter for modeling the chemical transfer between air and water [44,45]. It plays a crucial role in predicting the transport, behavior, and fate of pesticides, especially semi-volatile organic compounds (SVOCs).

Since many pesticides move as vapor between the air and the soil, plant, and water surfaces, the direction of this movement is controlled by the HLC [46]. Goodarzi et al. [47] said that a pesticide’s HLC is the main parameter that determines how likely it is to change from a liquid form to a gas form. In contrast, gaseous-to-liquid partitioning is essential in describing the associations between pesticides and rain, cloud water, fog water, or the alveoli of human and animal lungs [48]. Spencer et al. [49] indicated that chemicals with low HLC tend to accumulate at the soil surface when water is evaporating, resulting in increased volatilization with time. However, Feigenbrugel and Le Calve [50] concluded that, owing to the temperature dependence of HLC, the oxidation of the pesticides (for example, fenpropidin and pyrimethanil) in the gas and liquid phase can compete. Consequently, the pesticide has a high tendency not to be persistent in the atmosphere in comparison to in soils. 

The octanol–water partition coefficient (K_ow_), which measures a pesticide’s tendency to partition between water and lipids, is also an essential factor in volatilization. Low K_ow_ pesticides are more water-soluble and less likely to evaporate, whereas high K_ow_ pesticides are more lipophilic and more likely to evaporate [51]. The soil adsorption coefficient (K_oc_) also influences volatilization, as it dictates the pesticide’s binding strength to soil particles. Pesticides with a high K_oc_ are more likely to adsorb to soil and reside there, reducing their volatilization potential. 

The soil–air partition coefficients (K_soil-air_) are a significant factor in determining the transport and fate of airborne pesticides in the environment. The K_soil-air_ value is dependent on a number of variables, including the physicochemical properties of the pesticide, soil characteristics, and environmental conditions. According to Davie-Martin et al. [52], K_soil-air_ value can vary significantly based on soil composition and environmental conditions (such as temperature and humidity), influencing the degree of pesticide volatilization from soil to air. Pesticides with high K_soil-air_ values are more likely to volatilize and be transported by air, potentially exposing to humans and contaminating the environment. Wu [53] projected the K_soil-air_ values of some POPs, such as HCH, DDE, DDD, and dichlorodiphenyltrichloroethane (DDT) in cultivated soil in different climatic scenarios. The reductions in K_soil-air_ values of the studied pesticides imply less capacity of the soil to absorb these compounds, resulting in more significant volatilization of these compounds to the atmosphere.

To sum up, pesticide properties such as vapor pressure, molecular weight, water solubility, K_ow_, and K_oc_ significantly affect the air dispersion process. These characteristics of commonly used pesticides are displayed in Table 2. The environmental persistence and formulation, as well as the application and environmental conditions, play a significant role in minimizing volatilization and the potential for environmental contamination or off-target dispersal. 

### 3.3. Pesticide Applications, Spray Drift, and Volatilization Process

Farmers encounter significant challenges, notably the risk of low crop yields caused by threats from pests, weeds, and diseases that can potentially decimate entire crops. By employing pesticides judiciously, farmers can effectively manage these challenges, resulting in improved crop yields and enhanced food security. While various spraying technologies have been developed to optimize crop production, it is imperative to assess their potential environmental and health impacts, particularly the heightened risk of air volatilization. 

Volatilization is the transformation of pesticides from a liquid or solid state to a gaseous state, enabling their dispersion into the atmosphere [35]. This process has significant implications for air quality, as volatile pesticides can travel long distances and contaminate non-target areas, posing risks to human health, wildlife, and the environment. Pesticides are typically applied to plant foliage through spraying, and during this process, varying proportions of the spray unintentionally intercept the crop canopy and/or bare soil surface. As a result, the most probable fate mechanisms for pesticides include surface runoff, volatilization, movement with eroded soil particles, and photodecomposition [83].

Pesticide spray drift is the phenomenon that can lead to pesticide volatilization to the non-target areas during pesticide application to fields. Pesticide droplets deflect out of the canopies and are transported by the wind to locations other than the targeted areas [4,84]. Even though an effective pesticide spray application is used to achieve the required level of pest control, a portion of the pesticide spray may be lost to the ground and air [85]. Under conditions of stable air application, off-target drift may still penetrate or reach non-target plants and environmental media. As a result, the phenomenon potentially impacted human health and polluted the environment [36,86]. 

In general, droplet size governs the competition between absorption by plant surfaces and volatilization. Small droplets tend to evaporate faster than larger ones, which tend to be absorbed [7]. However, many recent studies have focused on the transport of pesticide droplets as influenced by weather conditions and product types to adjacent areas rather than on the effect of spraying conditions [87,88]. Pezzi and Rondelli [89] studied the outlet air angle and fan speed in an air-assisted sprayer. The effect of airflow on pesticide losses was more evident than that of air jet direction. A study by Gil et al. [88] on normalized spray loss suggested that the outward flow is mainly influenced by droplet size and wind speed. 

Cross et al. [90,91,92] found that airflow rate and droplet size have an important impact on airborne spray drift, while the liquid flow rate of the spray did not show any effect on spray drift. This is similar to a finding of Ramaprasad et al. [85], who pointed out that sprayer systems designed to dispense a coarse droplet with a size >200 mm, can increase rapid contact of pesticides with the foliage or land surface. Moreover, Hewitt et al. [93] demonstrated that adjuvant use directly affects spray break-up through some common nozzle types, changing droplet size distribution and drift potential. Hewitt [94] explained that the transportation of pesticide sprays is primarily influenced by application conditions, especially nozzle design, size, angle, and air-to-liquid velocity ratios. Moreover, the physical properties of the tank mix can significantly affect the spray formation, spray quality, and evaporation of pesticides. 

During pesticide drift, which is also known as spray drift, a fraction of the amount applied to the target area could be deposited onto adjacent non-target areas and another fraction could be lost in the atmosphere [7,95,96]. Woodrow et al. [97] indicated that emissions represent a major dissipation pathway for most pesticides into the air, accounting for 50% or more of the amount applied. The movement and quantity of pesticides to a target area depend on a combination of several factors, such as the amount of pesticide sprayed, the physicochemical properties of the pesticide, the nozzle and operating pressure of the equipment, the height at which the pesticide is released, and climate conditions at the time of application, etc. [7,88,98,99]. However, Polyrakis [7] pointed out that the particle size distribution of the pesticide sprayed is perhaps the most important factor contributing to off-target drift. 

Post-application volatilization is just as important as chemical and microbiological degradation in moving a compound from a compartment (treated soil or plant surfaces) into the atmosphere several days or weeks after application [85,96]. Although many studies have indicated that the volatilization of pesticides to the soil is a major source of emission into the atmosphere, the rate of volatilization from plants seems to be higher [13,41,100]. Sarigiannis et al. [16] reported that volatilization from plant surfaces could be up to three times higher than soil. The volatilization process can be more dominant for total emissions of active substances than spray drift in the long run. After volatilization, these pesticides can be transported horizontally for long distances and generally undergo little mixing or dilution. If pesticides remain in the lower troposphere, local transport of pollutants in the range of 10 km is governed by the environmental conditions of the application area. However, if they are rapidly transported to the mid- and upper troposphere in the range of 5–16 km, their residence times increase with the range [101]. The transport time of an air parcel during large-scale vertical perturbations from the surface to a height of 10 km is measured in hours [102]. With the volatilization process, the amount of pesticide available to control pests and the potential for groundwater contamination decrease. 

### 3.4. Dispersion and Volatilization of Pesticide to Atmosphere

Apart from volatilization, pesticides can also enter the atmosphere through degradation pathways (hydrolysis in water and soils, photolysis, and reaction with OH radicals in the atmosphere), and wind erosion [4]. During the pesticide degradation process, the chemical transformation of pesticides into different compounds can play a role in the transfer of pesticides into the atmosphere [36]. Chemical reaction, photolysis, and microbial degradation are the three main types of degradation processes. Each of these processes is affected by a variety of factors, including the chemical structure of the pesticide, the conditions of the environment, and the presence of other substances. Pesticide transport includes volatilization, runoff, and leaching, while pesticide degradation includes photolysis, chemical reactions, and microbial breakdown. These behaviors are influenced by climatic conditions and climate change [36].

Pesticides can be degraded through chemical degradation in the soil [103]. Moreover, because it is constantly active, the photoreaction by sunlight radiation (photolysis) plays a significant role in the degradation of molecules on soil surfaces [104]. The rate and characteristics of chemical degradation are influenced by soil temperature, pH, soil moisture, and pesticide binding [105]. The degradation of pesticides through photolysis, a process triggered by the adsorption of light by the pesticides, influences the transfer of pesticides from aquatic systems to the atmosphere [106]. The rate of a pesticide’s photolysis is contingent on a number of factors, including its chemical structure, water pH, type of solvent, the amount of light present, the depth of the water, and the presence of other dissolved substances [107]. Rapid photolysis of pesticides can produce volatile byproducts, which can then be released into the air. 

The volatilization of pesticides from soils under dry conditions (water content below the permanent wilting point) could be significantly influenced by sorption to hydrated mineral surfaces. This sorption process strongly depends on temperature, pesticide properties, and soil properties [108,109,110,111]. In the field, it has been observed that many soils rapidly form a dry surface layer that greatly suppresses pesticide volatilization. The effect of soil drying is largely reversible; however, volatilization resumes when the soil is remoistened. Thus, any climatic condition or tillage practice that affects the soil moisture distribution will have a profound effect on the amount of volatilization [112,113,114].

The changes in volatilization correlate with the humidity-dependent sorption mechanisms of soil. Schneider et al. [110] studied the influence of different humidity regimes on the volatilization of two pesticides (triallate and trifluralin) from bare soil surfaces. They found that in very dry conditions, increasing the relative humidity in the adjacent air from 60 to 85% resulted in an up to 8 times higher volatilization rate of the pesticides. Another study by Wang et al. [114] showed that the distribution and persistency of the fumigant dimethyl disulfide (DMDS) were negatively correlated with soil moisture content. As indicated above, pesticides rapidly vaporize more from wet than from dry soils. Wolters [101] ascribed this phenomenon to the displacement of pesticides from soil surfaces by water due to increasing vapor pressure. However, Martinez et al. [115] reported no significant relationship between different moisture levels and sulfentrazone degradation in the soil, agreeing with the results of Chowdhury et al. [112] on atrazine degradation. This may be attributed to soil microbial activity, resulting in accelerated pesticide degradation [116].

The extent of pesticide adsorption also depends on soil organic matter content, type and amount of clay, ion exchange capacity, and pH [117]. Chowdhury et al. [112] observed that the persistence of atrazine and trifluralin in clay loam soil could last a few years, depending on temperature and soil organic matter, while a similar result on the persistence of DMDS in soil was displayed by Wang et al. [114]. In addition, a correlation between levels of SVOCs in the soil and soil organic matter was reported by Riaz et al. [118]. They also found that the level of SVOCs in the air was highly influenced by mountain soil, followed by urban soil and glacial soil, respectively. The adsorption of pesticides by the soil organic matter could be associated with its hydrophobic nature, which decreases its bioavailability to soil microbes [119,120]. Microbial degradation occurs most rapidly in warm, and moist soil that contains readily decomposable organic matter, such as manure or fresh plant material [7].

Other studies on the effect of soil property on pesticide distribution have also been presented. For example, Spencer et al. [121] indicated that the bulk density or porosity of soil is important because of its influence on the pesticides’ vapor and non-vapor phase movement and its effect on water evaporation rates. Another factor that influences pesticide volatilization rates is soil temperature. An increase in temperature in a soil–pesticide system could lead to increased pesticide volatilization. However, an increase in temperature may also increase the drying rate of the soil surface. Hence, volatilization may increase or decrease depending on its effect on soil drying and vapor pressure [122]. A finding by Luo et al. [123] showed that higher concentrations of organochlorine pesticides (OCPs) were observed in humus layers than in mineral layers, while a sharp decrease in concentrations with depth occurred in mineral layers. Similar trends were also reported in other studies [124,125], suggesting that POPs were mainly stored in organic horizons. Additionally, the presence of adjuvants in pesticide formulation is also a factor that can reduce or increase pesticide volatilization [7]. Nevertheless, Das and Hageman [126] found that with temperatures below 24.5 °C, the volatilization of three semi-volatile pesticides (chlorpyrifos, pyrimethanil, and trifluralin) from the soil was greater in the presence of adjuvants, while the opposite was observed for temperatures above 24.5 °C.

### 3.5. Influence of Environmental Factors on Pesticide Dispersion

Pesticides in the air are strongly affected by rain, snow, wind, humidity, temperature, sunlight, and thermal inversions [127]. A higher air temperature, for example, could distinctly increase the volatilization rate because the vapor pressure of most pesticides, particularly SVOCs, is exponentially temperature-dependent [108,122]. It could also increase the movement of the chemical to the surface by diffusion or mass flow in evaporating water. In a study by Spencer and Cliath [128], about 3–4 times of vapor pressure of pesticides increased with an increase in temperature of 10 °C. Schweizer et al. [129] examined the effect of temperature on the volatilization of pesticides from plants and surfaces. They observed that higher temperatures increased volatilization for all compounds (parathion-methyl, bromoxynil, and fluroxypyr), with up to 86% for fluroxypyr. However, this may not always be the case since a high temperature is also associated with a high drying rate of the soil surface, possibly decreasing vapor density and resulting in less volatilization than at the lower temperature. There is already some evidence of lesser volatilization but faster degradation rates due to increased temperatures [130].

Next to temperature, wind speed is another factor contributing to the dispersion of the pesticide through spray drift. High wind velocity promotes an increased pesticide volatilization rate. Moreover, Tepper [131] pointed out that the wind directly influences the concentrations of pesticides in the air, the transported droplets deposition location, and the direction and distance of transport. Waymann and Rüdel [132] found that volatilization from soil increased from 12 to 31% of the application dose for lindane when wind velocity increased from 0.4 to 1.7 m/s. They also reported that volatilization of lindane from French beans over 24 h increased from 52 to 62% when a wind velocity increased from 0.4 to 2 m/s. Cessna [133] identified the amount of two herbicides (trifluralin and triallate) lost by wind erosion and found that about 1.4% of the total applied was lost. The mobility of pesticides through wind erosion can have serious implications for both the environment and human health. Interestingly, Zaller et al. [23] made a noteworthy discovery concerning certain pesticides, such as cycloate, DDT, HCB, and permethrin, which had not been approved for use in Austria for several decades. They proposed several hypotheses to account for this finding, including the possibility of illegal pesticide imports or the depletion of existing stocks by applicators. Additionally, they considered the use of officially unapproved pesticides under temporary emergency authorizations, particularly neonicotinoids. Pesticides with exceptionally long half-lives that can persist for decades after application were also suggested as a potential factor. Contamination originating from pesticide stocks stored on farms and non-agricultural sources contributing to ambient air pollution was considered. Furthermore, the migration of these pesticides from neighboring countries into the study areas due to wind was proposed as part of the intricate explanation for their presence.

Air humidity is also an important factor for soil volatilization because of its effect on the evaporation rate and the moisture content of the surface layer of soil. Van den Berg et al. [13] indicated that when a pesticide is applied to a crop at low air humidity, the plant surfaces will dry faster than those at higher air humidity. High air humidity can enhance pesticide volatilization from plant surfaces. However, in cases where the pesticide has already been adsorbed onto the plant surface, high humidity may impede its release into the air [100].

The frequency and intensity of rainfall, of course, have a role in the distribution of airborne pesticides through wet deposition. Increased rainfall will have consequences on the amount of pesticide transport processes. Wetter soils have higher hydraulic conductivities and thus pesticide-rich water can transport more rapidly vertically and horizontally inside the soil matrix and be available for microbiological degradation [108]. Under intense convectional storms, surface runoff could become more pronounced, but for typical advective storms, its significance might not be as substantial [134]. Like rain, snow also highly contributes to the wet deposition of pesticides. In addition to the low temperature, snow with a very high porous structure can collect these pesticides from the air in gas and particulate phases [135].

Thurman and Cromwell [136] determined triazine herbicides in rainfall on Isle Royale in Lake Superior. Analyzing the predominant wind direction indicated that the herbicides originated from the upper Midwest and were subject to long-distance transport before being deposited by rain. High concentrations of atrazine were observed in the surface layer of the lakes during deposition periods and decreased later in other seasons. Another study by Mast et al. [137] reported that atrazine, carbaryl, and dacthal were frequently detected in rain at concentrations substantially higher than in snow. The most frequently detected current-use pesticides (CUPs) in snow were dacthal, endosulfan, and chlorothalonil. This implies that cold condensation does not significantly influence pesticide distributions among studied areas compared to rain. Looking at pesticide application times and temperatures, pesticide concentrations in air and precipitation are much higher in the Spring and Summer, leading to increased re-volatilization from soil and plant surfaces [137,138].

Daly et al. [139] proposed that the presence of pesticides in the atmosphere within remote mountain regions is shaped by a combination of factors, including local sources, long-range transport, and cold-trapping mechanisms. On a related note, Ding et al. [140] conducted an investigation into the atmospheric distribution of CUPs (currently used pesticides) and OCPs (organochlorine pesticides) across four different mountains in Southern British Columbia, Canada, taking into account various environmental variables. Their findings revealed a notable pattern: OCP concentrations in the air were relatively consistent across all four mountains. In contrast, the concentrations of CUPs in the air tended to decrease as altitude increased, particularly in areas influenced by nearby ground-level sources. This suggests that the proximity of pesticide sources and altitude have a more significant influence on atmospheric pesticide distribution than factors like precipitation and vegetation cover.

## 4. Environmental and Human Health Consequences from Pesticides as Airborne Pollutants

### 4.1. Environmental Pollution from Airborne Pesticides

Under the scenario that only 1% of the pesticides applied to crops reach the target organism, this means 99% of them are deposited in non-target compartments. Subsequently, these pesticides can contaminate soil, air, surface water, groundwater, and sediments, as well as agricultural products via adsorption, leaching, volatilization, spray drift, and runoff processes [141,142]. In addition, different types of pesticides lead to different impacts on both environmental contamination and human health risk [36]. For example, OCPs, which have low acute toxicity, could accumulate in biological tissues, causing long-term damage in the food chain [143], while organophosphate pesticides (OPPs) lead to acute toxicity in mammals due to their low persistence [144]. 

**Figure 1:**
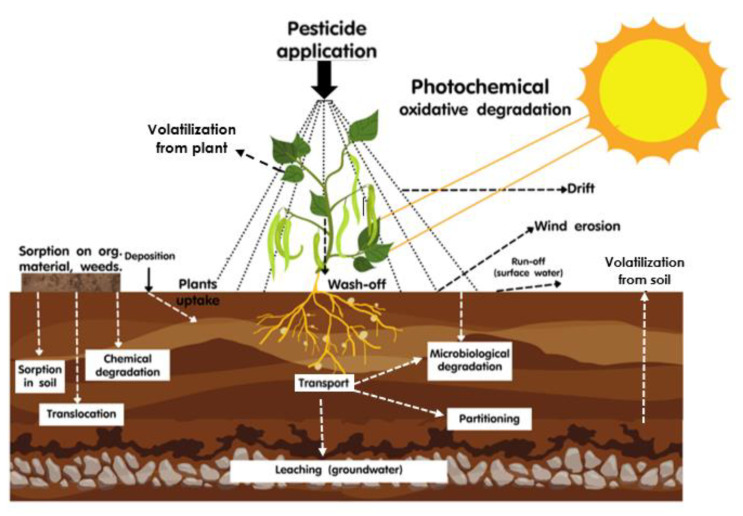
Dispersion pathway of pesticides in the ecosystem and their volatilization through plants and soil process (modified from Galon et al. [122]).

Despite being banned or restricted in many countries, certain pesticides, notably OCPs, have still been detected in the environment [145]. This indicates that despite regulatory efforts, these persistent chemicals continue to enter the ecosystem through various sources. When pesticides are volatilized (Figure 1), they can travel a long distance far away from the source to the atmosphere [36], causing global contamination. Wong et al. [146] indicated that soils are important sinks and sources of persistent organic pollutants in the atmosphere. The dissipation rate of pesticides from tropical areas is often faster than in temperate climates due to increased volatility and degradation rates [147]. However, insecticides are less likely to volatilize when they adsorb to soil particles [146]. Delving into the realm of OCPs, a study by Terry et al. [148] sheds light on an ongoing phenomenon: soil samples from agricultural fields in the Fraser Valley and orchards in the Okanagan Valley, British Columbia, Canada, continue to release legacy pesticides into the regional atmosphere. This curious revelation is attributed to the transport of more volatile OCPs, such as HCH and HCB, across the Pacific Ocean from Asian countries to the western coast of North America, facilitated by atmospheric currents. In contrast, the persistence of DDT residues in the environment appears to be a result of a complex interplay between long-distance transport and local legacy sources. Furthermore, instances of pesticide contamination have emerged in pristine environments, including ice cores from alpine glaciers in Europe [149], high-altitude national parks situated above 2200 m in Brazil [150], the Bolivian Andes at elevations up to 5200 m [151], and even in insects collected from nature conservation areas in Germany [152].

Sultana et al. [153] evaluated the contamination status of OCPs and their associated potential for air–soil exchange from ecologically important sites of the Indus Basin, Pakistan. Among different OCPs investigated, DDTs and HCHs were more prevalent compounds in the agricultural soils and ambient air of the area. These compounds could pose a threat to the natural habitat and biodiversity of Indus Basin. A study by Qu et al. [154] exhibited that endosulfan, which is one of the dominant OCPs found in both soil and air samples of the Campanian Plain, Southern Italy, poses significant ecological risks to some terrestrial species.

Apart from soil–air exchange (Figure 1), Tudi et al. [36] stated that volatilization also affects the exchange of pesticides between air and water and that could be the major source of human exposure to OCPs contaminated surface water. Chakraborty et al. [155] reported that more than 90% of the atmospheric OCPs along the bank of the Hooghly River in India were in the gaseous phase. Rainfall and atmospheric depositions are the main pathways for OCPs to contaminate the surface water [147]. Khuman and Chakraborty [156] assessed the eco-toxicological risk of using pesticidal persistent organic pollutants in the lower stretch of the transboundary river Ganga, India and they observed that endosulfan can pose serious risks to the edible fish species in the river. Similarly, Kang et al. [19] reported that the air–seawater exchange of selected OCPs showed that OCPs tended to migrate from the atmosphere to the ocean and corals, increasing the environmental pressure on coral reef ecology. 

Spray drift is another airborne movement of spray droplets during pesticide application [36]. Pan et al. [157] indicated that about 2–25% of total pesticides are lost during drifting, thus, causing environmental pollution and food contamination [158,159]. For example, chlorpyrifos (ChF) can contaminate aquatic environments via many routes such as runoff, leaching, and spray drifts [160]. Farhan et al. [161] observed that sub-lethal concentrations of ChF can induce oxidative stress and histological alterations in the tissues of tilapia. Giddings et al. [162] suggested that the effects of ChF in aquatic systems significantly depend on the duration and magnitude of exposure and toxicity to individual species. Moreover, Yang et al. [163] highlighted pyrethroid pesticides, which can contaminate water due to leaching spray drift, making fish more susceptible to oxidative stress caused by environmental pollutants. This result agrees with Sumon et al. [164] who displayed that spray drift of OPPs indirectly affects algae and macrophytes, community metabolism, rotifers, and other macro-invertebrates in aquatic ecosystems of north-west Bangladesh. 

While numerous studies have highlighted the toxic impact of airborne pesticides on a range of organisms, including mammals, birds, fish, bees, and earthworms [165,166,167,168,169], it is important to note that these assessments provide a broad overview of potential toxic exposure rather than predicting a specific number of animal fatalities. For a more precise evaluation of risks, it becomes essential to combine field-specific, spatially referenced usage data with biodiversity monitoring, as suggested by Mesnage et al. [170].

### 4.2. Human Health Risk from Airborne Pesticide

The intensive use of pesticides leads to ubiquitous contaminations, not only in soils, water, and/or crops but also in the atmosphere. Exposure to airborne pesticides may lead to acute and chronic health effects in humans of all ages [171]. Individuals residing in agricultural regions face heightened exposure to airborne pesticides through inhalation, particularly from pesticide spray drift in urban areas, parks, and even within their own homes. Farmers and their families, in particular, are at a greater risk of pesticide exposure compared to the general population. Furthermore, when nursing mothers and pregnant women come into contact with airborne pesticides, there is a potential for their children to be exposed as well. Certain pesticides have the capability to traverse the placenta and reach the developing fetus in the womb, as well as pass through breast milk to the nursing infant [172].

According to Mannucci and Franchini [173], more than three billion people, largely in developing countries, have been exposed to air quality levels exceeding the safety standard guidelines. Although many epidemiological studies have reported adverse effects of pesticides on oncological and hematological morbidity, pulmonary dysfunction, and immune system deficiencies in both adults and children [174,175,176,177,178], very few studies have demonstrated the impact of airborne pesticide exposure on human health. This could be explained by the fact that exposure to airborne pesticides is very variable and relatively unknown. Also, it is not easy to distinguish pesticide effects from the synergistic influence with other products (e.g., tobacco smoking). 

Pesticide poisoning can lead to acute toxic effects, which manifest within a short time frame ranging from a few minutes to several hours [179]. Exposure to air contamination during pesticide pulverization is the primary cause of acute toxicity. The health risk of pesticide pulverization depends on various factors such as the properties and toxicology of pesticides, level of exposure, and environmental conditions [180,181]. The impact of poisoning affects peripheral muscarinic and nicotinic receptors, as well as the central nervous system [182,183,184]. Some studies have reported that individuals who encounter both direct and indirect exposure to airborne pesticides could face a spectrum of acute toxic effects, including wheezing, coughing, irritation of the respiratory tract, blood in the sputum [185], burning sensation in the eyes, blurred vision, skin irritation, excessive sweating, shortness of breath [186], chest pain, fatigue, headaches, dizziness, nausea and vomiting, nose bleeding, scratchy noses or throats, and nail lesions [187]. A study conducted by Zaller et al. [23] raised concerning findings regarding pesticide classification. Their research revealed that out of 67 detected airborne pesticides, half of them (36) were categorized as acutely toxic, 39% exhibited reproductive toxicity, 24% were classified as carcinogenic, and 10% were identified as endocrine-active compounds. These findings underscore the significant risks associated with airborne pesticide exposure to humans, particularly in agricultural regions. Evidence of these acute risks has been documented in various parts of the world, including the USA [188], Costa Rica [189], Italy [190], France [191], India [192], Malaysia [193], China [194], and South Africa [195].

Extensive data collected from laboratory animals provide strong support for the correlation between airborne pesticide exposure and the development of chronic health issues, but it should be noted that comprehensive epidemiological data are not available for all health conditions. Some documented evidence reveals a clear association between the most frequently detected airborne pesticide and the occurrence of various chronic diseases and disorders in humans. For example, Tsai et al. [196] indicated that airborne pesticides, focusing on OCPs, diakylphosphates, and pyrethroid may influence children’s growth during infancy or childhood and neurodevelopment. Guida et al. [197] also suggested that people, especially infants and toddlers, may be exposed to an increased risk of hepatic cancer through OCP inhalation. A study by Wang et al. [198] demonstrated that different cytotoxic effects on human hepatocellular live carcinoma cells (HepG2) and human skin keratinocyte cell line (KERTr) were found in both indoor and outdoor dust with OCPs accumulation. According to Rauh et al. [199], it has been observed that even extremely low doses of chlorpyrifos can result in brain abnormalities in fetuses and children. Additionally, in rats, exposure to chlorpyrifos at such low doses has been found to hinder locomotor activity, affect behavior, and disrupt neurotransmitter systems, as highlighted by Perez-Fernandez et al. [200].

Rudzi et al. [193] found that occupational exposure to CUPs led to significant contamination of the blood serum among Malaysian farmers, in stark contrast to non-farmers. Doǧanlar et al. [201] discovered that, intriguingly, individuals living in agricultural regions of Turkey, even without occupational exposure, displayed elevated pesticide concentrations in blood in comparison to residents in the control area. According to Tang et al. [202], there is a noteworthy association between soil p,p′-DDE (the primary bioactive component in DDT) and breast cancer. This link was particularly pronounced in several major agricultural regions of China, where relatively higher levels of DDT were detected. Niu et al. [203] exhibited that the Chinese inhabitants who live in agricultural regions or areas with higher soil organic matter or soil with pH < 7, were also exposed to higher carcinogenic risks of DDTs from the soil. 

In a study by Gascon et al. [204], the prenatal exposure of individuals was assessed through cord blood tests measuring DDE and HCB. The study reported the presence of asthma in participants up to the age of 14 years and discovered a significant correlation between prenatal exposure to these substances and adverse effects on the respiratory system. Karmaus et al. [205] explained that DDE could alter the immune system’s response by promoting the production of Th2 lymphocytes, thereby contributing to the development of allergic diseases like asthma. Moreover, the association between DDE levels in the blood and lung function was examined by Balte et al. [206]. The findings showed that DDE had an adverse effect on German children’s height, subsequently affecting their forced vital capacity and forced expiratory volume in one second.

Raherison et al. [207] reported a notable association between the presence of ethylene thiourea in urine, which is a byproduct of the degradation of certain fungicides, and the occurrence of asthma and rhinitis in French children. Their results also showed that children who live in vineyard rural areas are at a higher risk of airborne dithiocarbamate (insecticide) exposure during the summer period. This is in agreement with a finding of Suarez-Lopez et al. [208] implying that pesticide spray seasons could amplify the likelihood of pesticide exposure among young children who are not involved in agricultural activities. Furthermore, these exposures appear to have a temporary impact on blood pressure, leading to short-term increases.

Due to their toxic nature and the prolonged exposure experienced by individuals, either willingly or inadvertently, these airborne pesticides not only are directly associated with an increased incidence of cancer [209] but also are suspected to disrupt the endocrine and immune systems [210]. While the precise mechanisms underlying these health impacts are not yet fully understood, compelling evidence suggests that pesticides induce disruptions in enzymatic function and signaling mechanisms at the cellular level. DNA-based toxicity research further indicates that airborne pesticides may have an impact on gene expression, potentially leading to epigenetic changes that can be inherited across generations [172]. Moreover, they pose a risk to the central nervous system and are implicated as potential factors in the development of Alzheimer’s and Parkinson’s diseases [211,212]. 

## 5. Mitigating the Risks to the Environment and Human Health Associated with Pesticide Spraying Methods

**Figure 2:**
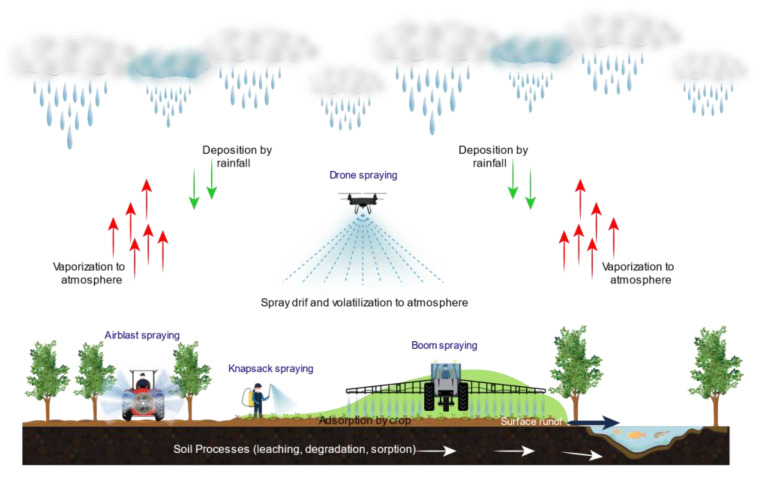
The impact of spraying techniques on spray drift and atmospheric volatilization.

The selection of pesticide spraying methods plays a crucial role in determining the extent of risks posed to environmental and human health, as well as crop production costs. Each method possesses distinct characteristics that influence the potential for environmental contamination, encompassing factors like volatilization, spray drift, and impacts on neighboring areas (Figure 2). These methods differ in their implications on human health, encompassing aspects such as worker exposure and the likelihood of unintentional exposure to nearby individuals. Moreover, the choice of spraying methods can have financial implications, with factors such as equipment needs, labor requirements, and pesticide consumption affecting production costs. 

**Figure 3:**
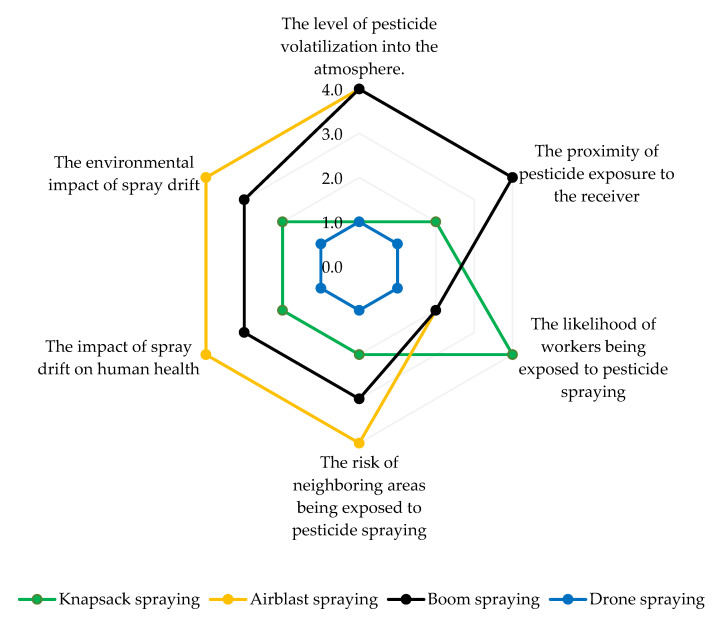
Spider chart of spraying methods’ impact on environmental and human health. Level of impact: 0 = No incidence; 1 = Low incidence; 2 = Middle low incidence; 3 = Middle high incidence; 4 = High incidence.

This section evaluates the environmental and human health impacts of the most frequently used pesticide spraying methods. To accomplish this, a comprehensive assessment was conducted using a spider chart (Figure 3). The provided ratings aim to offer a general understanding of the relative impact associated with these methods, and they should be interpreted as indicative rather than definitive or precise. Based on the chart, it is evident that the degree of pesticide volatilization is influenced by the spraying method employed and can originate from both soil and plant surfaces. For example, airblast spraying, characterized by the generation of a fine mist, typically carries a higher likelihood of volatilization compared to methods like knapsack spraying (Figure 2). This is due to the finer droplet size and increased surface area exposed to air, which enhances the potential for pesticide volatilization. Aircraft serve as vital aerial spraying platforms for crop protection, offering substantial payload capacity and operational efficiency. However, they are susceptible to pesticide drift, where droplets can deviate from their intended path due to factors like aircraft wingtip vortices, even in the absence of wind. This phenomenon becomes pronounced when pesticides are released at high flight altitudes, leading to the creation of wake vortices that can capture and transport pesticide droplets over long distances. Such spray drift poses environmental and health risks, potentially harming nearby crops and becoming a significant concern for both users and regulatory bodies. Consequently, there has been a growing interest in the use of agricultural multi-rotor unmanned aerial vehicles (UAVs), commonly known as drones or remotely piloted aircraft (RPA), as novel sprayers in recent years. Drone spraying (Figure 2) with its precision and targeted application, has the potential to mitigate the risk of volatilization by minimizing the release of pesticides into the air. Utilizing drone spraying technology (Figure 2) offers a promising solution for reducing the risk of volatilization by enabling precise and targeted pesticide application, minimizing the dispersion of pesticides into the air. It is crucial to emphasize that the operation of such drones should strictly adhere to guidelines provided by regulatory bodies like the US Environmental Protection Agency (EPA). Moreover, effective crop protection drones must be equipped with advanced features such as high-precision GPS systems and sophisticated software programming. These enhancements play a critical role in not only reducing volatilization but also mitigating spray drift, ensuring the efficient and responsible application of pesticides.

When considering the pesticide exposure distance to the receiver, which refers to how far pesticides can potentially travel from the application site, methods that generate a fine mist and/or apply pesticides from a height or over a large area, such as airblast or boom spraying, generally result in a larger exposure distance (Figure 2). This is because the fine droplets produced by these methods can be carried by air currents and dispersed over a wider range. In contrast, methods like knapsack spraying, which involve closer proximity to the application site and produce larger droplets, may result in a relatively smaller exposure distance. 

Spraying workers, particularly those using methods like knapsack spraying that require direct handling and close proximity to the pesticide application, may face higher risks of exposure. The proximity to the spray and potential inhalation or contact with the pesticides can increase the likelihood of adverse health effects. The use of drone spraying offers the potential to mitigate risks for workers as they can control the application remotely, reducing direct exposure to pesticides. 

The impact on neighboring areas or other potential impacts focuses on the likelihood of pesticides affecting areas beyond the intended target. Airblast spraying or boom spraying, which cover a large area and/or create a fine mist, may have a higher chance of impacting neighboring areas. This can occur through spray drift, where pesticide particles are carried by wind or air currents beyond the targeted area. The smaller droplet size and increased potential for airborne transport in these methods contribute to a higher risk of spray drift. Conversely, methods like knapsack spraying, which involve more localized application, may have a lower likelihood of impacting neighboring areas.

Given the potential ramifications for both human health and the environment, spray drift is an important factor to consider. Methods that generate a fine mist like airblast spraying, offer the advantage of facilitating high hourly performance and precise application at optimal stages for effective pest and disease control, resulting in reduced water consumption and production costs. However, this method is more prone to drift, which can lead to unintended exposure to pesticides for nearby individuals and wildlife. Knapsack spraying, especially when not accompanied by appropriate precautions such as shielding or wind speed management, can also pose significant risks of spray drift. This spray drift not only imposes a financial burden on agriculture farmers but also results in suboptimal outcomes. In contrast, the use of smart technology like drone spraying, with its precise and controlled application, offers the potential for reduced risk of spray drift, as the targeted release of pesticides can minimize the dispersion of particles beyond the intended area. 

Drone spraying offers a range of advantages, including cost-effective and user-friendly equipment, as well as the ability to fly at a consistent speed and altitude, and apply precise spray quantities of pesticides. Its operational efficiency exceeds manual spraying by over tenfold, while operators can remotely control the process, enhancing safety by reducing pesticide exposure and conserving agricultural water. Moreover, drone-based monitoring systems provide vital insights into water systems, soil conditions, pest presence, and fungal infestations. These systems offer regular, real-time updates, enabling farmers to maintain continuous oversight of crop yields. This uninterrupted access to up-to-date crop information empowers farmers to make informed decisions promptly, optimizing their crop management practices and taking corrective actions as needed.

The choice of spraying methods can have significant implications for pesticide volatilization, exposure distance, worker safety, impact on neighboring areas, and spray drift. Understanding these factors helps inform decisions and practices to minimize the potential risks associated with pesticide spraying while ensuring effective pest and disease control in agricultural and horticultural practices. 

## 6. Effective Strategies for Minimizing Pesticide Exposure

**Figure 4:**
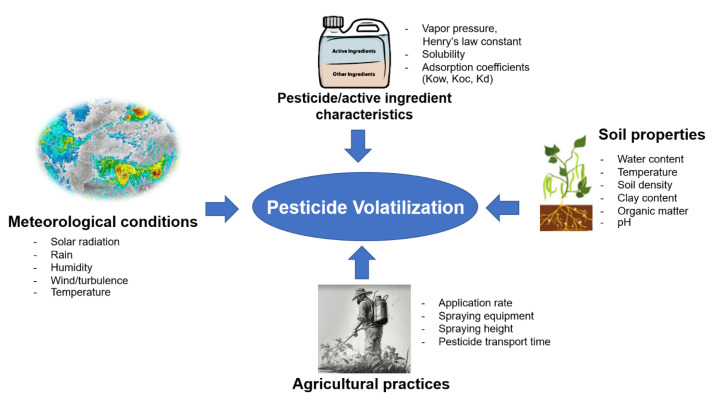
Key factors involved in the volatilization process causing pesticide dispersion to the atmosphere.

Despite advances in our understanding of pesticide volatilization and spray drift as a result of this review, additional research is necessary to address the complex challenges posed by these phenomena. Figure 4 demonstrates the key factors involved in the volatilization process causing pesticide dispersion into the atmosphere. The agricultural practices involved with environmental conditions during pesticide spraying as well as pesticide and soil properties are the major parameters. Consequently, better knowledge of the fate and transport of airborne pesticides is essential for accurate risk assessment and management. To reduce the negative effects of airborne pesticides, research is needed on innovative technology for safer application, education programs for responsible usage, and stronger policies for sustainability and environmental protection. This collective approach will safeguard ecosystems and public wellbeing.

The scientific literature reviewed in this study clearly indicates that the main pathways pesticides get into the air are through volatilization, spray drift, and soil emissions. Volatilization can be especially important for pesticides with high vapor pressures, while spray drift can be important for pesticides that are used in large amounts. To minimize the risks associated with volatilization and spray drift, research for engineering design of the low-drift spray nozzles and the appropriate droplet sizes to reduce the risk of pesticide drift and subsequent human exposure is needed. The favorable weather conditions (e.g., low wind speeds) in applying pesticides is also a critical issue that can limit widespread pesticide dispersion. Intensive research in landscape management such as establishing buffer zones between treated fields and sensitive areas (e.g., residential zones, water bodies, schools) help is required to reduce pesticide drift and protect susceptible populations from exposure. 

Pesticide contamination of the environment depends on factors such as pesticide properties, application methods, and local environmental conditions. Understanding these influences is crucial in developing effective and sustainable pest management strategies that reduce environmental and health risks associated with pesticide use. Integrated pest management (IPM) offers a comprehensive approach, incorporating non-chemical methods like biological control, cultural practices, and pest-resistant plant varieties to minimize reliance on chemical pesticides and reduce the likelihood of airborne pesticide exposure. Further research into agroecological practices, such as crop diversification and conservation tillage, can enhance pest resistance and decrease pesticide usage. A robust monitoring and surveillance program is vital for tracking airborne pesticide levels, identifying areas of concern, and monitoring trends over time. Implementing a combination of these strategies will mitigate the risks associated with airborne pesticide exposure and promote safer and more responsible practices in agriculture and beyond, fostering a healthier and more sustainable environment.

To significantly reduce the risks posed by pesticide volatilization and spray drift, it is crucial to develop and implement effective policies and regulations. Future research should evaluate the efficacy of existing policies and identify areas for improvement, such as stricter guidelines for pesticide registration, mandatory buffer zones, and application restrictions under certain weather conditions. Additionally, greater international cooperation is required to harmonize pesticide regulations and promote the global adoption of best management practices. The research can aid in the development of stricter guidelines for pesticide registration, use, and management, as well as the implementation of best management practices to reduce pesticide drift. Effective communication strategies to increase public awareness of the risks associated with airborne pesticide exposure and promote sustainable agricultural practices are currently much needed research. This involves collaborating with stakeholders, such as farmers, policymakers, and the public, to reduce airborne pesticide risks. 

Education and training are the most essential strategies for mitigating the dangers posed by airborne pesticides. By providing farmers and pesticide users with the necessary knowledge and training on proper pesticide handling, storage, and application techniques, the risk of pesticide drift and associated exposure could be reduced [213]. Implementing stringent regulations on the registration, use, and monitoring of pesticides and encouraging the adoption of best management practices can contribute to the reduction of pesticide exposure in the air. Encouraging farmers and pesticide users to use appropriate personal protective equipment can protect them from direct exposure to airborne pesticides. Lastly, increasing public awareness of the risks associated with exposure to airborne pesticides and encouraging community participation in monitoring and reporting pesticide use can promote responsible pesticide management and reduce exposure risks.

**Figure 5:**
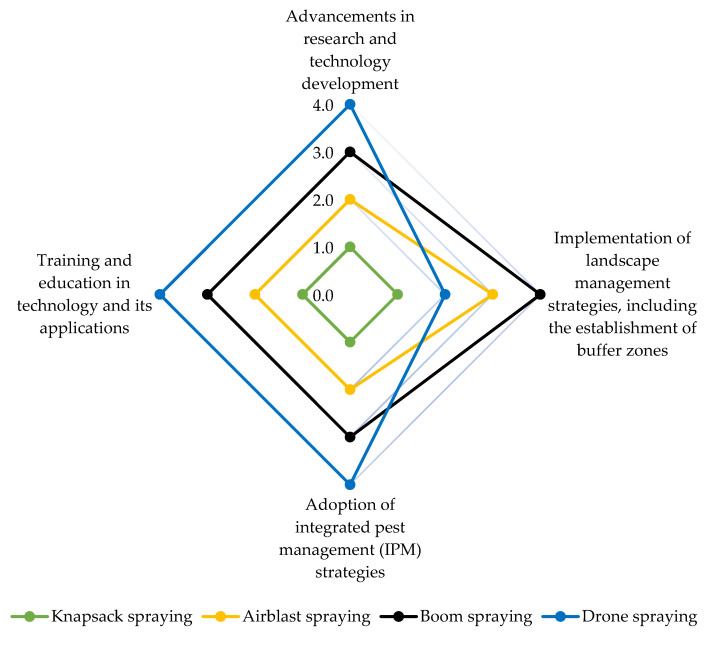
Spider chart of provisional cost analysis for each mitigation method aimed at reducing environmental and health risks. Level of cost: 1 = Low cost; 2 = Middle low cost; 3 = Middle high cost; 4 = High cost.

This review assesses the preliminary cost analysis of various mitigation strategies intended to reduce the environmental and health risks associated with airborne pesticides, taking into account the unique characteristics of each spraying method. This analysis aids in making informed decisions regarding the selection and adoption of mitigation measures, ensuring the effective management of pesticide-related risks while considering the financial aspect associated with different spraying methods. It can be seen that the research and development costs associated with different spraying methods vary (Figure 5). For knapsack spraying, the costs might be relatively low since the technology is simple. However, there may be expenses associated with developing and testing new low-drift nozzles or formulations. 

In the case of airblast spraying, mitigating measures could involve research and development costs for making and testing sensor technology to enable more precise application, advanced spray technology, and new low-drift nozzles. Boom spraying technology is well-established, but research and development costs may arise from the development of new mitigation measures such as advanced spray technology, shielded booms, and precision agriculture techniques. These costs could include equipment design, materials testing, and field trials. On the other hand, drone spraying, being a relatively new and complex technology, may incur high research and development costs due to software development, rigorous testing to meet regulatory standards, and the integration of precise application techniques. 

When considering landscape management costs in relation to different spraying methods, knapsack spraying is commonly employed in smaller and more controlled environments or for spot treatments. As a result, the costs associated with landscape management, such as the establishment and upkeep of buffer zones, are generally lower compared to other methods. Airblast spraying is often utilized in orchards or vineyards, where the landscape tends to be more structured and potentially easier to manage. However, due to the characteristics of the spray, it may require significant landscape management efforts, including the maintenance of buffer zones and windbreaks. The efforts entail additional costs but are indispensable for mitigating potential environmental impacts. Boom spraying, typically used in larger open-field environments, can incur high costs for landscape management. Managing buffer zones and vegetation in such expansive settings can be both costly and complex. Precision agriculture techniques like GPS can assist in optimizing the application process, but they also introduce additional costs. As for drone spraying, while it enables precision application, it may still necessitate the establishment of buffer zones, particularly near sensitive habitats or water sources. However, due to the precise nature of drone spraying, the costs associated with landscape management may be lower compared to other methods. 

The implementation costs associated with IPM in knapsack spraying are likely to be lower compared to other methods, primarily due to its smaller scale and localized application. However, successful IPM requires regular pest monitoring and potentially the utilization of various pest control methods tailored to the specific situation. Although airblast spraying can be more targeted than boom spraying, it may require significant investment for IPM implementation. This includes the incorporation of sensor technology for precise application and the development of appropriate pest-monitoring programs. These investments are crucial for effective pest management and minimizing environmental impacts. Implementing IPM in boom spraying can involve some costs, particularly in adopting advanced technologies like GPS for precision spraying. While implementing IPM with drone spraying can be initially costly due to the need for high-tech equipment, specialized training, and sophisticated software for precision spraying, the precise application enabled by IPM can result in long-term cost savings by reducing pesticide usage and optimizing pest control outcomes for both boom spraying and drone spraying.

When training costs are considered, it could be observed that knapsack sprayers are generally easier to operate compared to other mentioned systems, which may result in lower training costs. However, training is needed to ensure safe and efficient usage, as well as proper techniques to minimize drift and maintain the equipment. Training for airblast spraying should focus on the unique aspects of this equipment, including the correct adjustment and operation of the sprayer, as well as strategies to minimize off-target movement. It may involve costs associated with gaining a comprehensive understanding of airblast spraying techniques. Boom spraying systems can be complex, requiring comprehensive training covering various topics such as equipment calibration, proper operation, maintenance, and adaptation to different weather conditions and crop types. Due to the breadth of knowledge required, training for boom spraying may result in additional costs. Drone spraying is likely to require the most extensive and costly training due to the intricacies of drone operation, adherence to drone-specific regulations, and proficiency in utilizing precise application technologies. The expenses involved may include professional drone operator certification and specialized training in pesticide application using drones.

The cost analysis indicates that each spraying method has its own considerations for minimizing pesticide exposure. Knapsack spraying may have the lowest overall cost, while airblast spraying, boom spraying, and drone spraying may require higher investment costs due to their technological advancements. It is important to weigh these costs against the potential benefits in terms of reduced environmental and health risks associated with pesticide application. 

## 7. Conclusions

This critical review emphasizes the global presence of pesticides in the atmosphere and delves into key factors influencing their dispersion into the air. These factors include pesticide properties, application methods, volatilization, and other dispersion pathways, along with their environmental and health impacts. To effectively address the associated risks, a comprehensive and interdisciplinary approach is paramount, considering recent advancements in science, technology, policy, and agricultural practices. Enhanced understanding of airborne pesticide fate and transport is crucial, alongside the development of novel formulations and application technologies, improved predictive modeling, sustainable farming practices, and robust policies and regulations. Collaboration among researchers, policymakers, and stakeholders is vital to make progress and ensure responsible pesticide use in agriculture. Moreover, the complex interplay of factors, such as rapid global population growth, demographic shifts favoring an aging population, climate change, and increasing carbon dioxide emissions, significantly shapes the landscape of airborne pesticides. These dynamics affect food production, alter pesticide usage patterns, influence labor demand in agriculture, and potentially pose health risks, particularly among the elderly. Adapting to these evolving challenges in agricultural practices and pesticide management is imperative to mitigate risks, promote sustainable food production, and safeguard human health.

## Figures and Tables

**Table 1:** Concentration of pesticides in atmosphere.

Pesticide	Name	Concentration Range (ng/m^3^)	Country	References
Organochlorine Insecticide	Aldrin	0.36–1.18	China	[17]
	*cis*-Chlordane	0.01	South Africa	[18]
	trans-Chlordane	0.02	South Africa	[18]
	Chlordane	nd ^1^–1.369	China	[19]
	2,4′-DDD	0.01	South Africa	[18]
	4,4′-DDD	0.37–0.55	France	[20]
		0.011–0.087	Pakistan	[21]
		4.57–154	China	[17]
		0.02	South Africa	[18]
	2,4′-DDE	0.02	South Africa	[18]
	4,4′-DDE	0.27–0.36	France	[20]
		0.002–0.007	China	[22]
		2.05–25.60	China	[17]
		0.012–0.240	Pakistan	[21]
		1.20	South Africa	[18]
		11.60–109.50	Austria	[23]
		nd–0.20	USA	[24]
	2,4′-DDT	0.06	South Africa	[18]
	4,4′-DDT	0.1	South Africa	[18]
		0.009–0.068	China	[19]
		12.50–15.50	Austria	[23]
	Dieldrin	0.08	South Africa	[18]
	Drins	nd–0.01	China	[19]
	α-Endosulfan	0.37–81.31	France	[20]
		0.011–0.028	China	[22]
		0.52–1.09	China	[17]
		0.00009–0.042	China	[19]
		0.003–0.009	Pakistan	[21]
		0.06–0.10	South Africa	[18]
	HCB	0.05–0.43	USA	[24]
		nd–0.026	China	[19]
	α-HCH	0.0058	South Africa	[18]
	β-HCH	0.0027	South Africa	[18]
	δ-HCH	0.0005	South Africa	[18]
	γ-HCH	0.06	South Africa	[18]
	HCHs	0.00012–0.0184	China	[19]
		0–267.4	Germany	[25]
	Heptachlor	0.0015	South Africa	[18]
	Heptachlor epoxide	0.00065	South Africa	[18]
	4,4′-methoxychlor	0.0004–0.0088	China	[19]
	Mirex	0.00013	South Africa	[18]
		nd–9.94	USA	[24]
	Oxychlordane	0.00048	South Africa	[18]
Organophosphate Insecticide	Azinfos-methyl	0.03	South Africa	[18]
	Chlorpyriphos	10–12900	Republic of Korea	[26]
		0.11–0.15	Spain	[15]
		0.10–1.00	South Africa	[27]
		6.10–36.10	Costa Rica	[28]
		15.50–287.0	Austria	[23]
		16.20	South Africa	[18]
	Diazinon	0.28–1.49	France	[20]
		1.40	South Africa	[18]
	Dimethoate	0.19	South Africa	[18]
		0.10–1.00	South Africa	[27]
	Ethoprophos	0.21–0.48	France	[20]
	EPN	0–4470	Republic of Korea	[26]
	Malathion	0.10–1.00	South Africa	[29]
		0.20	South Africa	[18]
	Parathion	1000–61900	Republic of Korea	[26]
	Phorate	2100–33700	Republic of Korea	[26]
Carbamates	Carbaryl	1.30	South Africa	[18]
	Oxamyl	9.64	Spain	[30]
Acetamides	Metazachlor	0.009	South Africa	[18]
		0.17–3.13	France	[16]
	S-metolachlor	0.29	South Africa	[18]
Triazinones/ Triazines/ Triazoles	Atrazine	0.04	South Africa	[18]
	Metribuzin	0.03	South Africa	[18]
	Simazine	0.88	South Africa	[18]
	Terbuthylazine	0.79	South Africa	[18]
	Propiconazole	0.08	South Africa	[18]
Herbicide	Alachlor	0.12–6.03	France	[20]
	Alconifen	0.23–4.15	France	[16]
	Diuron	0.12	South Africa	[18]
	Dimethenamid	0–1556.6	Germany	[25]
	Glyphosate	503.0–517.0	Malaysia	[31]
		0.24–0.48	USA	[32]
		0.18–1.04	France	[33]
		20.3–3176.8	Germany	[25]
		0.10–0.30	Italy	[34]
	Metolachlor	0–1273.3	Germany	[25]
		12.3–382.6	Austria	[23]
	Pendimethalin	0–3916.8	Germany	[25]
		44.9–3932.4	Austria	[23]
	Prosulfocarb	13.7–4357.8	Austria	[23]
	Terbuthylazine	0–905.9	Germany	[25]
	Trifluralin	0.12–40.74	France	[16]
Fungicide	Captan	1.19–67.62	France	[20]
		4.54–22.82	France	[16]
	Chlorothalonil	0.11–107.93	France	[20]
		0–1866.2	Germany	[25]
		30.6–554.4	Austria	[23]
	Carbendazim	0.010–0.046	Spain	[15]
	Difenoconazole	77.43	Spain	[30]
	Epoxiconazole	0.12–3.99	France	[20]
		0–81.3	Germany	[25]
	Folpet	7.90–82.2	France	[16]
		0–7613.8	Germany	[25]
		35.5–1665.2	Austria	[23]
	Hexachlorobenzene	0–46.3	Germany	[25]
	Tebuconazole	22.2	South Africa	[29]
		10.4–67.7	Austria	[23]
	Tetraconazole	11.1–16.3	Austria	[23]

^1^ nd = not detected.

**Table 2:** Pesticide properties related to dispersion and volatilization in atmosphere.

Pesticide	Molecular Weight (g/mol)	Boiling Point (°C)	Density (g/cm^3^)	Log K_ow_^1^	Log K_oc_^2^	Water Solubility (mg/L)	Vapor Pressure (Pa)	Henry’s Law Constant (Pa.m^3^/mol)	Reference
Acetamides	59.07–292	N/A ^3^	0.85–2.9	−1.52	1.15–3.3	10–99,500	N/A ^3^	N/A ^3^	[54,55]
Alachlor	269.8	108–109	1.14	3.36	2.91	191	0.17	2.24	[56]
Alconifen	328.9	400	-	6.2	-	0.03	1.8 × 10^−9^	2.46	[57]
Aldrin (as Cl)	364.9	175–177	1.57	5.2	5.3	Insoluble	0.027	0.697	[58]
Atrazine	215.7	N/A ^3^	1.19–1.5	2.83	1.5–2.77	33–200,000	0.0004–0.4	3–110	[59,60,61]
Azinfos-methyl	214.6	96–98	1.3	2.3	2.36	1200	0.02	0.016	[62]
Captan	300.35	Decomposes	1.77	2.98	3.1	4.4	2.6 × 10^−8^	N/A ^3^	[63]
Carbamates	86–389	N/A ^3^	0.97–1.6	−6.9	0.6–3.9	0.1–63,000	N/A ^3^	N/A ^3^	[64,65]
Carbaryl	201.2	142–143	1.23	1.88	1.94	42	0.0001	0.0005	[66]
Carbendazim	191.2	Decomposes	1.47	2.26	1.8	48.3	2.1 × 10^−7^	N/A	[67]
α-Chlordane	409.8	433–435	1.6	5.37	5.5	2.2	0.15	1.44	[58]
γ-Chlordane	409.8	433–435	1.6	5.37	5.5	2.2	0.15	1.44	[58]
Chlordane	409.8	433–435	1.6	5.37	5.5	2.2	0.15	1.44	[58]
Chlorothalonil	265.7	Decomposes	2.3	3.33	3.49	0.35	6.5 × 10^−8^	N/A ^3^	[68]
Chlorpyriphos	350.6	156–157	1.49	4.8	4.7	0.6	0.00011	0.00044	[69]
DDX	320.9	150–155	1.66	5.25	5.28	0.06	0.0013	1.32	[70]
2,4′-DDD	320.9	210–211	1.57	4.82	5.03	Insoluble	0.00011	0.0015	[71]
4,4′-DDD	320.9	210–211	1.57	4.82	5.03	Insoluble	0.00011	0.0015	[71]
2,4′-DDE	318.9	185–186	1.65	5.07	5.2	0.0017	0.00013	0.08	[71]
4,4′-DDE	318.9	185–186	1.65	5.07	5.2	0.0017	0.00013	0.08	[71]
2,4′-DDT	321	260–261	1.6	6.1	5.75	Insoluble	0.00005	0.0015	[72]
4,4′-DDT	321	260–261	1.6	6.1	5.75	Insoluble	0.00005	0.0015	[72]
Diazinon	304.3	83–84	1.17	3.7	3.45	5.5	0.013	0.011	[69]
Dichlorvos	220.5	96–98	1.44	1.82	2.08	700	0.01	0.014	[62]
Dieldrin	380.9	385–386	1.7	4.64	4.85	0.05	0.0029	0.0057	[72]
Dimethoate	229.7	86–88	1.33	1.79	1.93	2000	0.13	0.038	[62]
Diuron	233.22	Decomposes	1.31	2.47	2.66	14.8	3.8 × 10^−10^	2.7 × 10^−8^	[73]
Endosulfan	406.9	408–409	1.86	4.8	4.97	0.03	0.00015	0.00038	[72]
Epoxiconazole	430.8	135–143	1.25	4.26	3.99	0.23	0.0018	3.3 × 10^−6^	[74]
Ethoprophos	240.3	88–90	1.34	2.63	2.68	16.2	0.04	0.023	[62]
Folpet	240.3	155–157	1.98	0.47	N/A ^3^	2.2	N/A ^3^	N/A ^3^	[74]
Glyphosate	169.07	Decomposes	1.7	−4.5	−4.1	1.7	2.1 × 10^−9^	N/A ^3^	[75]
HCB	284.8	288.5	1.3	4.06	4.09	0.16	0.0048	0.0089	[72]
β-HCH	290.8	288–289	1.3	4.29	4.34	Insoluble	0.00039	0.00068	[72]
γ-HCH	290.8	288–289	1.3	4.29	4.34	Insoluble	0.00039	0.00068	[72]
α-HCH	290.8	288–289	1.3	4.29	4.34	Insoluble	0.00039	0.00068	[72]
Heptachlor	373.4	205–208	1.5	5.21	5.28	Insoluble	0.0026	0.058	[72]
Heptachlor epoxide	389.8	370–380	1.8	4.87	4.94	Insoluble	0.00016	0.00027	[72]
4,4′-methoxychlor	345.9	105–110	1.4	4.18	4.32	Insoluble	0.0027	0.0021	[72]
Malathion	330.3	156–157	1.19	2.91	3.01	17.5	0.0005	0.0013	[62]
Metazachlor	283.8	N/A ^3^	1.34–1.36	4.03	N/A ^3^	26–3800	0.005–0.2	1.5–22	[76,77]
Metribuzin	214.7	298.5	1.43–1.46	1.79	1.47	14–15,000	0.0069	0.52–2.1	[78]
Mirex	545.5	600–610	3.1	5.5	5.89	Insoluble	0.00025	0.00045	[72]
Oxychlordane	409.8	250	1.5	5.16	5.22	Insoluble	0.0002	0.00038	[72]
Parathion-methyl	263.8	150–152	1.23	3.84	3.54	8.7	0.019	0.065	[62]
Propiconazole	342.2	195–196	1.28	4.4	-	0.88	0.000013	1.24	[79]
S-metolachlor	345.9	N/A ^3^	1.4	4.25	N/A ^3^	5.5–11,000	0.013–0.05	0.7–37	[77]
Simazine	201.7	225.6	1.32–1.44	2.68	1.4–2.8	35–102,000	0.0003–0.06	2–53	[59,60]
Tebuconazole	307.8	130–133	1.25	3.94	3.67	68.8	0.00012	1.31	[80]
Terbuthylazine	285.3	135	1.28	2.68	2.32	60	0.00026	1.67	[81]
Trifluralin	335.84	120–123	1.4	6.24	4.9	0.2	5.5 × 10^−7^	N/A ^3^	[82]

^1^ Log K_ow_ = The log of a measure indicating how a chemical divides between water and fat-like substances. It indicates how readily a chemical can accumulate in organisms. ^2^ Log K_oc_ = The log of a measure showing how a chemical binds to soil or sediment versus staying in water. It hints at a chemical’s mobility in the environment. ^3^ N/A = Not available: Data are either not applicable or not provided for this field.

## Data Availability

Data will be made available on request.
